# Is neoadjuvant chemotherapy followed by surgery the appropriate treatment for esophagogastric signet ring cell carcinomas? A systematic review and meta-analysis

**DOI:** 10.3389/fsurg.2024.1382039

**Published:** 2024-05-06

**Authors:** Sabine Schiefer, Nerma Crnovrsanin, Eva Kalkum, Johannes A. Vey, Henrik Nienhüser, Ingmar F. Rompen, Georg M. Haag, Beat Müller-Stich, Franck Billmann, Thomas Schmidt, Pascal Probst, Rosa Klotz, Leila Sisic

**Affiliations:** ^1^ Department of General, Visceral and Transplantation Surgery, University Hospital Heidelberg, Heidelberg, Germany; ^2^ Department of Pathology, Netherlands Cancer Institute (NKI), Amsterdam, Netherlands; ^3^ Study Center of the German Society of Surgery (SDGC), University Hospital Heidelberg, Heidelberg, Germany; ^4^ Institute of Medical Biometry (IMBI), University of Heidelberg, Heidelberg, Germany; ^5^ Department of Medical Oncology, National Center for Tumor Diseases (NCT), University Hospital Heidelberg, Heidelberg, Germany; ^6^ Department of Visceral Surgery, University Center for Gastrointestinal and Liver Diseases, St. Clara Hospital and University Hospital, Basel, Switzerland; ^7^ Department of General, Visceral, Cancer and Transplant Surgery, University Hospital Cologne, Cologne, Germany; ^8^ Department of Surgery, Cantonal Hospital Thurgau, Münsterlingen, Switzerland

**Keywords:** esophagogastric neoplasm, esophagogastric cancer, signet ring cell carcinoma (SRCC), neoadjuvant chemotherapy, systematic review, meta-analysis

## Abstract

**Background:**

The impact of neoadjuvant chemotherapy (nCTX) on survival and tumor response in patients with esophagogastric signet ring cell carcinoma (SRCC) is still controversial.

**Methods:**

Two independent reviewers performed a systematic literature search in Medline, CENTRAL, and Web of Science including prospective and retrospective two-arm non-randomized and randomized controlled studies (RCTs). Data was extracted on overall survival (OS) and tumor regression in resected esophagogastric SRCC patients with or without nCTX. Survival data was analyzed using published hazard ratios (HR) if available or determined it from other survival data or survival curves. OS and histopathological response rates by type of tumor (SRCC vs. non-SRCC) were also investigated.

**Results:**

Out of 559 studies, ten (1 RCT, 9 non-RCTs) were included in this meta-analysis (PROSPERO CRD42022298743) investigating 3,653 patients in total. The four studies investigating survival in SRCC patients treated with nCTX + surgery vs. surgery alone showed no survival benefit for neither intervention, but heterogeneity was considerable (HR, 1.01; 95% CI, 0.61–1.67; *p* = 0.98; *I*^2 ^= 89%). In patients treated by nCTX + surgery SRCC patients showed worse survival (HR, 1.45; 95% CI, 1.21–1.74; *p* < 0.01) and lower rate of major histopathological response than non-SRCC patients (OR, 2.47; 95% CI, 1.78–3.44; *p* < 0.01).

**Conclusion:**

The current meta-analysis could not demonstrate beneficial effects of nCTX for SRCC patients. Histopathological response to and survival benefits of non-taxane-based nCTX seem to be lower in comparison to non-SRC esophagogastric cancer. However, certainty of evidence is low due to the scarcity of high-quality trials. Further research is necessary to determine optimal treatment for SRCC patients.

**Systematic Review Registration:**

https://www.crd.york.ac.uk/, PROSPERO (CRD42022298743).

## 1 Introduction

Gastric cancer is the fourth common malignancy and caused about 769.000 cancer related deaths worldwide in 2020 (1). Until today radical surgery—combined with other treatment modalities if necessary—remains the only curative treatment for gastric cancer. Various studies have shown an advantage of perioperative chemotherapy on survival compared to upfront surgery for esophagogastric cancer (2–4).

Incidence of gastric cancer has been slowly declining over the last years but in contrast to this overall reduction the incidence of signet ring cell carcinoma (SRCC) is increasing (5). According to the World Health Organization (WHO) definition SRCC is a subtype of poorly cohesive adenocarcinoma with more than 50% of signet ring cells (6). It was reported that SRCC is associated with younger age and female gender. Various meta-analyses have shown a worse prognosis for SRCC compared to non-SRCC (nSRCC) patients, especially for locally advanced SRCC (7–9).

Neoadjuvant treatment such as chemotherapy and chemoradiotherapy increases survival in esophagogastric adenocarcinoma (2, 3, 10, 11). However, most studies did not investigate the impact of neoadjuvant therapy on SRCC in a subgroup analysis. Hence, the prognostic impact of nCTX on survival and tumor response in patients with esophagogastric SRCC is still controversial as previous studies yielded conflicting results (12–14). However, a comprehensive meta-analysis examining this topic is currently lacking in the literature. Aim of this study is to summarize the currently available evidence comparing OS and histopathological response rate after nCTX + surgery vs. surgery alone specifically in esophagogastric SRCC patients.

## 2 Methods

This systematic review and meta-analysis was carried out in accordance to the PRISMA guidelines (15) and in accordance with recommendations specifically for surgical systematic reviews (16). The study was conducted according to and registered at PROSPERO (CRD42022298743). There was no external source of funding.

### 2.1 Systematic literature search

A systematic literature search was performed in MEDLINE (via PubMed), Web of Science and Cochrane Central Register of Controlled Trials (CENTRAL) on 29th September 2022 (17). The following search strategy was performed for MEDLINE:

((“Signet Ring Cell”[tiab] OR “Signet Ring Cells”[tiab] OR “Signet Cell”[tiab]) AND (cancer[tiab] OR carcinoma*[tiab] OR adenocarcinoma*[tiab] OR neoplas*[tiab] OR tumor[tiab] OR tumors[tiab] OR tumour*[tiab] OR malignan*[tiab])) OR “Carcinoma, Signet Ring Cell”[Mesh].

AND

(neoadjuvant*[tiab] OR neo-adjuvant[tiab] OR preoperativ*[tiab] OR pre-operative[tiab] OR perioperativ*[tiab] OR peri-operative[tiab] OR “followed by”[tiab] OR following[tiab] OR “Neoadjuvant Therapy”[tiab] OR “Neoadjuvant Therapy” [MeSH].

AND

radiochemotherap*[tiab] OR chemoradiotherap*[tiab] OR chemoradiation*[tiab] OR chemotherap*[tiab] OR “Chemoradiotherapy”[Mesh]) OR “Induction Chemotherapy”[Mesh].

The full search strategies for the other databases are available in the Supplementary material.

Additionally, a hand search through references of relevant studies was performed.

### 2.2 Study selection

Randomized and non-randomized, prospective and retrospective two-arm studies, including patients with esophagogastric adenocarcinoma with signet ring cells treated with neoadjuvant or perioperative chemotherapy followed by surgery compared to surgery alone (nCTX + surgery vs. surgery), were eligible for inclusion. All studies investigating survival or histopathological response to nCTX in adenocarcinoma including signet ring cells were included. Studies with exclusively adjuvant chemotherapy, any chemoradiotherapy or radiotherapy were excluded. Animal studies, meeting abstracts, letters, comments, editorials, publications for which the full text is irretrievable and non-English studies were also excluded.

Titles and abstracts were reviewed independently by two reviewers (SS, NC) to select full papers for further evaluation. If there were disagreements additional reviewers (LP, RK) were consulted. Any disagreement was resolved by consensus.

### 2.3 Data extraction and statistical analysis

Data was extracted independently by two reviewers using a standardized form composed prior to data extraction. The following items were extracted: title, first author, year of publication, country, study period, sample size, type of chemotherapy, tumor localization, stage, definition of SRCC, survival outcomes and histopathological response data.

If there was a training and a validation set, the data of the validation set was extracted. If hazard ratios (HR) were not explicitly reported the HR was estimated by the formulas proposed by Tierney et al. (18). The original survival curves were extracted using WebPlotDigitizer (19) to calculate HR.

Primary statistical analysis and meta-analysis were performed with RStudio, version 2023.03.1 + 446 using the package meta (20, 21). A random-effects model was used to account for methodological and clinical heterogeneity. Statistical heterogeneity among the effect estimates of the included trials was evaluated using the *I*^2^ statistic and *τ*^2^. According to Cochrane heterogeneity was interpreted as follows: 0%–40% low, 30%–60% moderate, 50%–90% high, and 75%–100% considerable. The HR and its standard error were used as effect measure for OS. Data was pooled using the inverse-variance method. The response rate was pooled as odds ratio (OR) with 95% CI using the Mantel–Haenszel (M-H) method (22). The results were graphically illustrated by forest plots.

### 2.4 Critical appraisal (bias)

For randomized studies, the risk of bias and quality was assessed by using the Cochrane Collaboration tool for assessing risk of bias 2.0 (23). The tool includes five standard domains of bias: “bias arising from the randomization process”, “bias due to deviations from intended interventions”, “bias due to missing outcome data”, “bias in measurement of the outcome” and “bias in selecting of the reported result”. These domains were rated as high risk of bias, low risk of bias, some concerns or unclear. Finally, an overall risk of bias was evaluated.

For non-randomized studies, the risk of bias assessment was conducted in accordance to ROBINS-I tool (24). Each of the following domains were evaluated: “bias due to confounding”, “bias in selection of participants into the study”, “bias in classification of intervention”, “bias due to deviations from intended interventions”, “bias due to missing data”, “bias in measurement of outcomes”, “bias in selection of the reported result”. Each potential source of bias was graded as low risk, moderate risk, serious risk, critical risk or no information/unclear. An overall judgement of the presence of bias in each study was made.

Furthermore, for each outcome the certainty of the evidence was rated to be very low, low, moderate, or high for each outcome using the GRADE system (25). This includes limitations in the design from the risk of bias assessment, indirectness of evidence, unexplained heterogeneity or inconsistency of results, imprecision of results, and publication bias.

## 3 Results

### 3.1 Study selection

**Figure 1:**
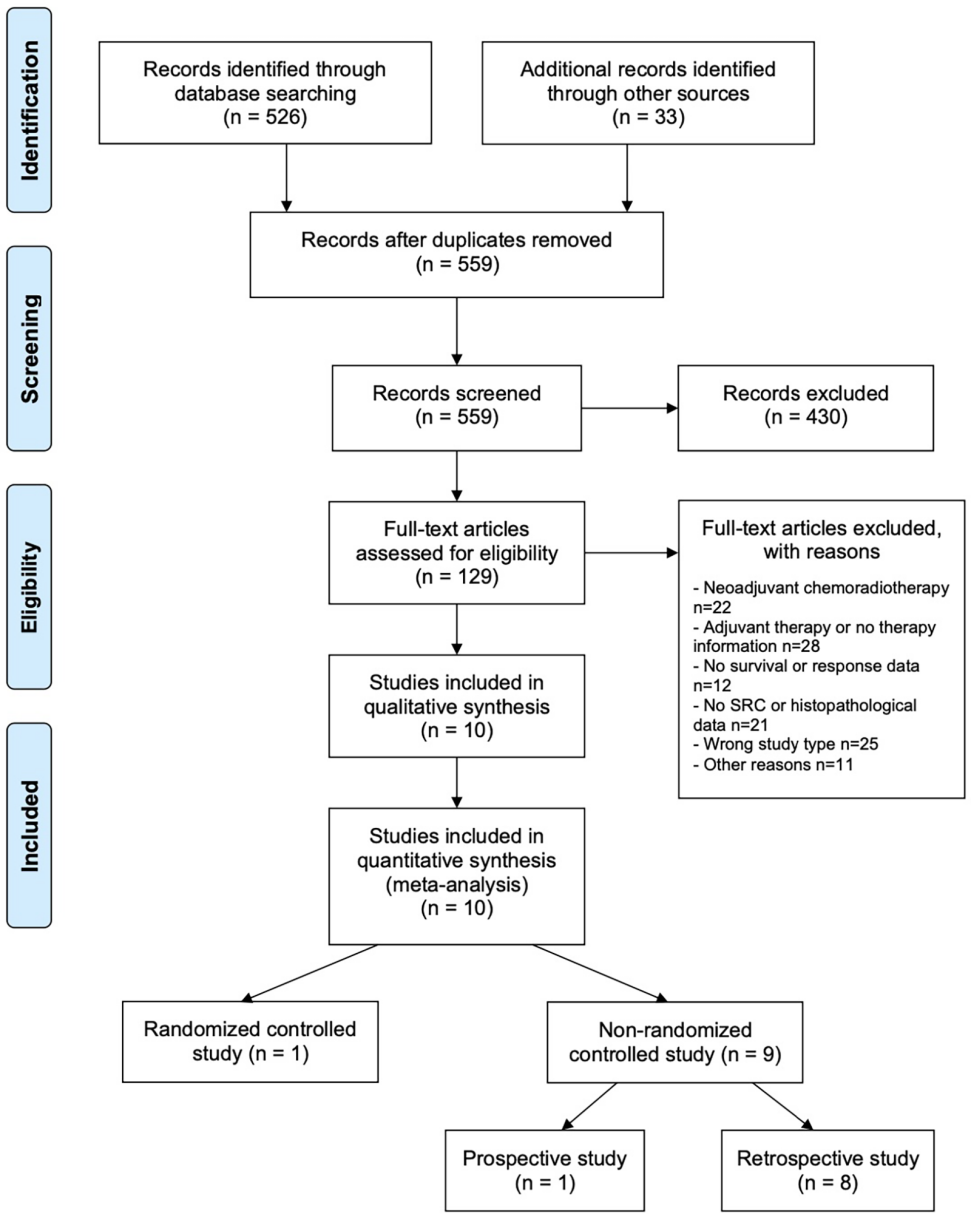
PRISMA flow chart of study selection.

526 studies were identified by database search, 33 additional sources were identified by screening the references of reviews and meta-analysis of current esophagogastric cancer literature, resulting in a total of 559 articles screened for this review. 129 of these articles meet the inclusion criteria and were evaluated in full-text. From these, 119 trials were excluded because of nSRCC examination or missing histology data (*n* = 21), wrong study type (*n* = 25), neoadjuvant chemoradiotherapy (*n* = 22), adjuvant chemotherapy or no information about treatment (*n* = 28), missing data (*n* = 12) and other reasons (*n* = 11). Ten studies were finally included for meta-analysis. A PRISMA flow chart of study selection is shown in Figure 1. One study was a RCT (26). Nine studies were non-RCTs with one prospective and eight retrospective analyses (12, 13, 27–33). Of ten studies six were from European and four from Asian countries. Three studies (12, 29, 30) analyzed locally advanced cancer patients whereas all other studies analyzed patients at all tumor stages.

A summary of the included studies is shown in Table 1.

**Table 1:** Summary of studies eligible for meta-analysis: sample size, treatment regiments, localization, stage, definitions of SRCC and TRG, and reported outcome.

Study type	Author (year)	Country	Period	*N*	nCTX + surgery vs. surgery (%)	nSRCC vs. SRCC (%)	nCTX	Tumor localization	Stage	Definition of SRCC	TRG system	Reported outcome
nCTX + surgery vs. surgery												
RS (12)	Heger et al. (2018)	Germany	2002–03/2016	283	172/111 (60,8/39,2)	–	EOX, FLOT, 24% others	E, EGJ, S	cT3/4 any NM	SRC containing		OS: HR, 0.5; 95% CI, 0.37–0.68
RS (13)	Messager et al. (2011)	France	1997–2010	924	171/753 (18,5/81,5)	–	ECF, FU + platinum, 18.5% others	S	I–IV	>50% SRC		OS: calculated HR, 1.24; 95% CI, 1.04–1.49
RCT (26)	Iwasaki et al. (2021)	Japan	10/2005–07/2013	176	89/87 (50,6/49,4)	–	SC	S	I–IV	SRC containing		OS: HR, 1.16; 95% CI, 0.81–1.67
RS (27)	Li et al. (2020)	China	2010–2017	72	36/36 (50/50)	–	SOX, DOS, XELOX, FOLFOX6, DOX	S	IIb–IVa	>50% SRC		OS: HR, 1.71; 95% CI, 0.76-3.84
SRCC vs. nSRCC												
RS (28)	van Hootegem et al. (2019)	Netherlands	2000–2016	298	–	234/64 (78.5/21.5)	carbo-/cisplatin + paclitaxel, cisplatin + 5-FU ± docetaxel, ECF	E, EGJ	any TNM	SRC containing	–	OS: HR, 1.36; 95% CI, 0.97-1.90
RS (29)	Schmidt et al. (2014)	Germany	1987–2010	714	–	504/210 (70.6/29.4)	cis-/oxaliplatin + 5-FU *1: +/− anthracycline/taxane	E, EGJ, S	cT3/4, *2 cN+	>50% SRC	–	OS: calculated HR, 1.44; 95% CI, 1.10–1.89
RS (30)	Xu et al. (2019)	China	05/2012–12/2017	264	–	176/88 (66.7/23.3)	SOX, XELOX	S	II–III	unclear	Mandard	OS: HR, 1.57; 95% CI, 1.09–2.26; HP response: OR, 0.26; 95% CI, 0.13–0.51
RS (31)	Heger et al. (2014)	Germany	1987–2011	692	–	470/222 (67.5/32.5)	+platinum/+epirubicin/+taxane/others	EGJ, S	any TNM	SRC containing	Becker	HP response: OR, 0.49; 95% CI, 0.33–0.74
PS (32)	Jary et al. (2014)	France	05/2011–01/2013	27	–	20/7 (74.1/25.9)	CEP	E, EGJ, S	T0–4, N0-3	unclear	Becker	HP response: OR, 0.12; 95% CI, 0.01–2.41
RS (33)	Jiang et al. (2021)	China	02/2016–12/2019	203	–	62/141 (30.5/69.5)	SOX	S	T2-4, *3 N−/+	>50% SRC	CAP	HP response: OR, 0.42; 95% CI, 0.22–0.81

The surgical procedure depended on localization of tumor. Five studies (13, 26, 27, 30, 33) analyzed gastric cancer patients. Iwasaki et al. as well as Jiang et al. used total gastrectomy ± lymphadenectomy (D2/D3) for resection. Li et al., Messager et al., Xu et al. used subtotal or total gastrectomy for resection ± lymphadenectomy (D1 or D2). Four studies analyzed patients with gastric cancer and carcinoma of the esophagogastric junction (EGJ) (12, 29, 31, 32). For all gastric cancer patients subtotal or total gastrectomy + D2 lymphadenectomy was used for resection. EGJ carcinoma were treated by transhiatal or abdominothoracic esophagectomy or transhiatal extended gastrectomy + lymphadenectomy (D1, modified D2 or D2).

One study (28) analyzed adenocarcinoma of the esophagus and esophagogastric junction but gave no detailed information about type of resection.

### 3.2 Neoadjuvant treatment

The chemotherapy regimens used in the neoadjuvant setting varied significantly and differed within some studies over time depending on new study results and recommendations. The majority of patients received a combination of platinum- and fluoropyrimidine-based CTX. In some studies triple chemotherapy with additional anthracycline and/or taxane was administered. Only a small fraction of patients (1.9%) received the FLOT regimen. An overview of the used regimens is given in Table 1.

### 3.3 SRCC definition

The definition of SRCC varied between the studies. Four studies used the WHO definition (>50% SRC) (13, 27, 29, 33), whereas four studies included patients with tumors containing SRC of any percentage (12, 26, 28, 31). Two studies (30, 32) gave no information for their SRCC definition.

### 3.4 Risk of bias within studies

One study (26) was rated using the Revised Cochrane risk-of-bias tool for RCTs. The overall risk was with some concern. All other nine studies (12, 13, 27–33) were rated using the ROBINS-I tool. Overall, the included studies had a low to moderate risk of bias. Due to the limited number of studies included in this meta-analysis assessment of the publication bias via funnel plot was not possible.

The risk of bias of each study for every domain is shown in Supplementary Tables S2, S3. Table 2 gives an overview of the overall risk for each study.

**Table 2:** Overall bias for all included studies.

Study	Author (year)	Overall bias
(12)	Heger et al. (2018)	moderate
(13)	Messager et al. (2011)	low to moderate
(26)	Iwasaki et al. (2021)	some concern
(27)	Li et al. (2020)	moderate
(28)	van Hootegem et al. (2019)	low
(29)	Schmidt et al. (2014)	moderate
(30)	Xu et al. (2019)	moderate
(31)	Heger et al. (2014)	low to moderate
(32)	Jary et al. (2014)	moderate
(33)	Jiang et al. (2021)	low

### 3.5 Overall survival

For this meta-analysis three analyses were performed.

**Figure 2:**
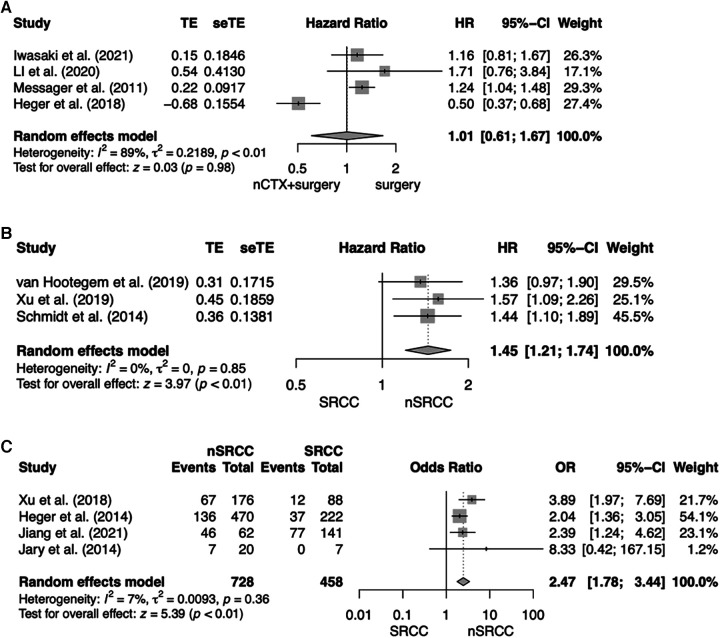
Forest plot for (**A**) OS for SRCC patients comparing nCTX + surgery vs. surgery; (**B**) OS after nCTX + surgery comparing SRCC vs. nSRCC patients; (**C**) Histopathological response after nCTX + surgery comparing SRCC vs. nSRCC.

The first analysis included four studies analyzing patients with SRCC comparing OS for nCTX + surgery vs. surgery alone (12, 13, 26, 27). The pooled HR indicated no beneficial effects on OS for neither intervention and marked heterogeneity between the studies (HR, 1.01; 95% CI = 0.61–1.66; *p* = 0.98; *I*^2 ^= 89%). Results are shown in Figure 2A. In total there were 1,455 patients, 468 patients (32.2%) were treated with nCTX + surgery and 987 patients (67.8%) underwent surgery only. Three primary studies indicated an OS benefit for surgery alone over nCTX + surgery whereas one study revealed a significant OS benefit for nCTX + surgery over surgery alone (12). The chemotherapy regimens used differed between and within the four studies (see Table 1).

Risk of bias assessment resulted in low to moderate risk of bias in one study, moderate risk of bias in two studies and some concerns in one study. The certainty of evidence was very low.

The second analysis compared three studies analyzing OS for patients treated by nCTX followed by surgery comparing SRCC to nSRCC patients (28–30). OS of SRCC patients was significantly worse compared to nSRCC patients (HR, 1.45, 95% CI = 1.21–1.74; *p* < 0.01; *I*^2 ^= 0%). For this analysis 1,276 patients were included, of which 362 patients (28.4%) had SRCC and 914 patients (71.6%) nSRCC. Two of the analyzed primary studies showed a significant OS benefit for nSRCC patients. The other study showed an OS benefit as well for nSRCC patients but was not significant. All results are shown in Figure 2B.

All but one study used chemotherapy regimens consisting of a platinum compound and a fluoropyrimidine, sometimes combined with a taxane.

Risk of bias assessment resulted in low risk of bias in one study and moderate in two studies. The certainty of evidence was very low.

### 3.6 Histopathological response

In the third analysis response rates were compared between patients treated by nCTX followed by surgery comparing SRCC patients to nSRCC patients (30–33). For this meta-analysis 1,186 patients were available, of which 458 patients (38.6%) were SRCC and 728 patients (61.4%) were nSRCC. Overall, histopathological response rates to nCTX were significantly higher in nSRCC patients (OR, 2.47; 95% CI, 1.78–3.44; *p* < 0.01; *I*^2 ^= 7%). Three of the included studies showed significantly higher histopathological response rates for nSRCC patients in comparison to SRCC patients. The fourth study yielded the same result, however, the difference in histopathological response between SRCC and nSRCC did not reach statistical significance. All results are shown in Figure 2C.

Two studies used platinum compounds and a fluoropyrimidine, two studies used a combination of platinum compounds plus a taxane and/or epirubicin for CTX treatment.

The four studies analyzing histopathological response rates used different tumor regression grading (TRG) systems. Response rate was 13.6% for SRCC vs. 38.1% for nSRCC in Xu et al., 16.6% vs. 28.9% in Heger et al., 54% vs. 74.2% in Jiang et al. and 0% vs. 35% in Jary et al.

Heger et al. and Jary et al. used the tumor response grading (TRG) system of Becker (34, 35) defining grade 1a + 1b as histopathological responders. Xu et al. used the Mandard-TRG (36) defining grade 1 + 2 with a tumor residuum of <10% as pathological responders. In contrast, Jiang et al. used the CAP-TRG (37) by the College of American Pathologist (38) and defined grade 0 + 1 + 2 as pathological responders.

Whereas histopathological response definition of Heger, Jary, and Xu et al. can be considered correspondent, Jiang applied a vaster definition of histopathological response including also residual cancer outgrown by fibrosis, which equals Mandard-TRG grade 3 + 4 (Supplementary Table S1). We therefore performed a sensitivity analysis without the study of Jiang et al. but yielded basically unchanged results (OR, 2.72; 95% CI = 1.56–4.76; *p* < 0.01; *I*^2 ^= 38%) (Supplementary Figure S1).

Risk of bias assessment resulted in low risk of bias in one study, low to moderate risk in one study and moderate risk in one study. The certainty of evidence was very low.

### 3.7 Risk of bias across studies

An overview of the rating of certainty of evidence was made with the grading of recommendations, assessment, development, and evaluation (GRADE) approach is shown in Table 3. The certainty of the evidence of the main analysis for all three outcomes was very low largely due to the study designs of the included studies (retrospective) and study biases.

**Table 3:** Certainty of the evidence for outcomes.

Outcomes	No. of included studies	Certainty of the evidence (GRADE)
OS nCTX + surgery vs. surgery	4	⊕◯◯◯ VERY LOW
OS SRCC vs. nSRCC	3	⊕◯◯◯ VERY LOW
Histopathological response	4	⊕◯◯◯ VERY LOW

## 4 Discussion

Various studies have shown an advantage of perioperative chemotherapy on survival compared to surgery alone for gastroesophageal cancer (2–4). Yet, pre- or perioperative chemotherapy for advanced SRCC is discussed controversially for possible chemoresistance and cancer progression during the preoperative regimen. Ultimately, the impact of perioperative chemotherapy on SRCC remains unclear, since the existing studies have yielded heterogeneous results.

This meta-analysis did not show a survival benefit for SRCC patients treated by nCTX + surgery compared to surgery alone. Yet, the number of existing studies investigating this topic is scarce and only four studies were found eligible for this meta-analysis. Messager et al. was the only study which reported a significant disadvantage for nCTX + surgery vs. surgery alone (HR = 1.24, 95% CI, 1.04; 1.48) (13). Iwasaki et al. and Li et al. presented a disadvantage for nCTX + surgery vs. surgery alone, which was not significant (HR = 1.16, 95% CI, 0.81; 1.67 and HR = 1.71, 95% CI, 0.76; 3.84) (26, 27). Heger et al. in contrast was the only study revealing a significant OS survival benefit for nCTX + surgery vs. surgery alone in SRCC patients (HR = 0.50, 95% CI, 0.37; 0.68) (12). However, the pooled HR of this meta-analysis have to be interpreted with caution and as an explorative estimate, since there was considerable heterogeneity (*I*^2 ^= 89%) between the studies, which might emerge from differences concerning CTX regimens, tumor stage, tumor localization, ethnicity, and SRCC definition.

One possible explanation for the divergent results is the type of chemotherapy used in the eligible studies. Chemosensitivity of signet ring cells could vary according to the drugs and combinations used. An ex vivo analysis of chemosensibility showed higher sensibility for docetaxel in diffuse and SRCC types compared to intestinal type gastric cancer cell lines (39). A study by Pernot et al. with 65 patients with advanced or metastasized SRCC showed a response rate of 65% and median OS of 14 months for patients treated with the TEFOX regimen (docetaxel + 5FU + oxaliplatin) and allowed a secondary resection in 40% of patients even in the metastasized patients (40).

Hence, whereas SRCC is thought to be generally less chemosensitive than nSRCC, it could rather have a specific sensitivity profile and be more sensitive to taxane-based chemotherapy (9). Causes can be multifactorial and are currently not well understood. One possible explanation could be a divergent tumor microenvironment causing unkwnown silencing enzymes that reduce cell death. Another reason might be a different tumor biology with a lower cell adhesion causing a reduced distribution of substances. However, evidence of specific prospective trials is lacking.

The study by Heger et al. was the only study with administration of FLOT-based CTX in 36% of the patients (*n* = 69), which is considered the current standard CTX-regimen for esophagogastric cancer (2, 41). In the study of Messager et al. the out-dated epirubicin-based regimen according to the MAGIC-trial or a fluoropyrimidine-/platinum-based therapy was applied in 82% of the patients, Iwasaki et al. treated his patients with S1 + cisplatin, and Li et al. presented various different regimens, but only 9 patients received a taxane-based CTX. Furthermore, Heger et al. included not only gastric but also junctional and esophageal cancer patients, whereas the other three studies included only gastric cancer patients.

Our meta-analysis demonstrated a significantly worse survival for neoadjuvantly treated SRCC patients compared to neoadjuvantly treated nSRCC patients. However, this does not necessarily mean, that perioperative CTX is not beneficial or even harmful for SRCC patients. Independent of the treatment strategy prognostic significance of SRC remains unclear, since study results have yielded divergent results (42–45). Previous meta-analysis found prognosis of SRCC to be stage-dependent (7, 8, 46). The result of our second meta-analysis is rather in line with these meta-analyses showing worse outcome for SRCC compared to nSRCC in advanced stages. Since neoadjuvant CTX is predominantly applied in locally advanced tumor stages, our results could reflect worse prognosis of advanced SRCC compared to nSRCC in general.

Most of the major esophagogastric cancer trials on pre- or perioperative multimodal treatment regimens—as for example MAGIC-, FFCD-, and CROSS-trial—are lacking information for SRCC or diffuse cancer (3, 4, 47). Only the FLOT4 study provided a subgroup analysis for SRCC and nSRCC and showed a non-significant survival benefit with FLOT compared to an epirubicin-based perioperative CTX, also for SRCC patients (2). In the light of these results and the limited existing evidence on perioperative CTX for SRCC, taxane-based perioperative CTX according to FLOT therefore should still be considered standard of care for locally advanced SRCC until further evidence proves differently.

Furthermore, our meta-analysis showed that the histopathological response rate after nCTx was significantly worse in SRCC patients compared to nSRCC patients. These results suggest a different chemosensitivity of signet ring cells to chemotherapy. As previously already discussed, the choice of chemotherapy regimen might influence chemosensitivity and hence response rates of SRCC. Bencivenga et al. suggested that tumors with different percentages of SRC might have a different response to chemotherapy (48). Hence, there could be a correlation between the fraction of SRC within a tumor and tumor response to chemotherapy. Unfortunately, the studies included in the meta-analysis used different tumor response grading systems and different definitions of histopathological response as described above. The more expanded definition of tumor response by Jiang et al. (33) explains the clearly higher response rates for SRCC and nSRCC in this study. However, conducting the meta-analysis without Jiang's study did not reveal any major changes in the results.

Limitations of our meta-analysis are the small number of included studies and the lack of data from RCTs. As almost all studies were retrospective and investigated mostly unmatched cohorts, overall quality of the included studies is poor with a relevant risk of bias. Furthermore, there was substantial heterogeneity across the studies regarding tumor localization and tumor stage as well as chemotherapy regimens as discussed above, limiting comparability of study results. These limitations delimitate the validity of our results restricting the applicability in clinical practice.

Another limitation is the inconsistent definition for signet ring cell cancer across the studies. As four included studies used the WHO definition of including patients with >50% signet ring cells in their histology but also four studies with any signet ring cell morphology. We agree with Mariette et al. that a concensus of the SRCC definition is crucial for further research. This research group suggests to name patients with >90% cohesive cells with signet ring cell morphology as SRCC. Histology with less than 90% signet ring cells should be named combined poorly cohesive (PC) not otherwise specified (NOS) and SRC carcinoma (PC-NOS/SRCC) or poorly cohesive NOS (PC-NOS) (49).

Patients with neoadjuvant chemoradiotherapy were excluded in this meta-analysis as we wanted to investigate the influence of exclusively chemotherapy on SRCC. However, a stronger response to chemoradiotherapy is possible, as a retrospective register study by Stessin et al. showed a better survival for cancer patients with gastric SRCC treated with adjuvant radiotherapy compared to without radiotherapy (HR, 0.71, *p* > 0.01) (50). Another study also showed a significantly improved median overall survival for SRCC patients when treated with adjuvant radiotherapy compared to surgery alone (33.0 months vs. 24.0 months) (51). However, data from the randomized ARTIST trial failed to show a survival benefit of adjuvant chemoradiation in patients with SRCC (52).

Currently, there is only one prospective, randomized study examining perioperative CTX compared to primary surgery followed by adjuvant chemotherapy in Caucasian patients with SRCC, the PRODIGE-19FFCD1103-ADCI002 trial by Piessen et al. (53). First results show a median OS of 39% for perioperative CTX compared to 28% for upfront surgery followed by adjuvant CTX (exploratory HR, 0.71, 95% CI, 0.40–2.64) for 83 eligible SRCC patients (14). However, final results are not expected before November 2027. Unideally in this study ECF is used as regimen, which may not be the optimal CTX in SRCC, since taxane-based regimens might be more effective (9, 40).

Research on targeted drugs in esophagogastric cancer patients is increasing, but insufficient regarding SRCC. The Checkmate 649 study showed improved survival for unresectable esophagogastric cancer patients (HR, 0.77, 95% CI, 0.64–0.92) when treated with nivolumab plus chemotherapy vs. chemotherapy alone but, did not include a subgroup analysis for diffuse cancer or SRCC (54). So far, we know that SRCC express programmed cell death ligand 1 (PD-L1) in 18.9%–60% (55–57). So SRCC patients may benefit from immunotherapy. Further research is much needed.

In conclusion, the optimal therapeutic strategy in SRC tumors remains unclear. This meta-analysis summarizes the available research on this topic and points out the lack of high-quality evidence. Further data, especially from RCTs is needed to allow more individualized treatment decisions in SRCC patients.

A taxane-based perioperative chemotherapy i.e., according to FLOT should still be considered standard of care for locally advanced SRCC until further evidence proves otherwise.

A standardized definition of SRCC as suggested by Mariette et al. (49) is mandatory to make study results comparable to optimize treatment recommentations for SRCC patients. Furthermore, a stratification according to SRCC and nSRCC in future studies is much-needed for better understanding of this tumor subtype and development of more tailored treatment approaches.

## Data Availability

The original contributions presented in the study are included in the article/**Supplementary Material**, further inquiries can be directed to the corresponding author.
