# Supplementary material for: Is neoadjuvant chemotherapy followed by surgery the appropriate treatment for esophagogastric signet ring cell carcinomas? A systematic review and meta-analysis
Source: Front Surg. 2024 May 6;11:1382039. doi: 10.3389/fsurg.2024.1382039 (PMC11102960; doi:10.3389/fsurg.2024.1382039)
Supplement: Supplementary file 1 [file Table1.docx]

Supplement Table 1: TRG classification system

| **Classification** | **Becker** | | **Mandard** | | **CAP** | |
| --- | --- | --- | --- | --- | --- | --- |
| TRG | 1a | No residual tumor | 1 | No residual tumor | 0 | No viable cancer cells  (complete response) |
|  | 1b | <10%  residual tumor | 2 | Rare residual tumor | 1 | Single cells or rare small groups of cancer cells  (near complete response) |
|  | 2 | 10-50%  residual tumor | 3 | Fibrosis outgrowing residual cancer | 2 | Residual cancer with evident tumor regression  (partial response) |
|  | 3 | >50%  residual tumor | 4 | Fibrosis outgrowing residual cancer | 3 | Extensive residual cancer with no evident tumor regression  (poor or no response) |
|  |  |  | 5 | Absence of regressive changes |  |  |
