# Supplementary material for: Is neoadjuvant chemotherapy followed by surgery the appropriate treatment for esophagogastric signet ring cell carcinomas? A systematic review and meta-analysis
Source: Front Surg. 2024 May 6;11:1382039. doi: 10.3389/fsurg.2024.1382039 (PMC11102960; doi:10.3389/fsurg.2024.1382039)
Supplement: Supplementary file 2 [file Table2.docx]

Supplement Table 2: Risk of bias for non RCT study using ROBINS-I tool

| **Study** | **Author (year)** | **Bias due to confounding** | **Bias in selection of participants** | **Bias in classification of intervention** | **Bias due to deviations from intended interventions** | **Bias due to missing data** | **Bias in measurement of outcomes** | **Bias in selection of the reported result** |
| --- | --- | --- | --- | --- | --- | --- | --- | --- |
| 12 | Heger, U (2018) | moderate | low | moderate | low | moderate | low | low |
| 13 | Messager, M (2011) | moderate | low | moderate | low | low | low | low |
| 27 | Li, Y (2020) | moderate | low | moderate | low | moderate | low | low |
|  |  |  |  |  |  |  |  |  |
| 28 | van Hootegem, S (2019) | low | low | low | moderate | moderate | low | low |
| 29 | Schmidt, T (2014) | moderate | low | low | low | moderate | moderate | low |
| 30 | Xu, X (2019) | moderate | low | low | moderate | moderate | low | low |
|  |  |  |  |  |  |  |  |  |
| 31 | Heger, U (2014) | moderate | low | low | low | moderate | low | low |
| 32 | Jary, M (2014) | moderate | low | low | moderate | moderate | moderate | low |
| 33 | Jiang, L (2021) | low | low | low | low | moderate | low | low |

Supplement Table 3: Risk of bias for RCT study using Revised Cochrane risk-of-bias tool for randomized trials (RoB 2)

| **Study** | **Author (year)** | **Bias due to  randomization process** | **Bias due to deviation from  intended intervention** | **Missing data  outcome** | **Bias due to  measurement of outcome** | **Bias due to selection  of reported results** |  |
| --- | --- | --- | --- | --- | --- | --- | --- |
| 26 | Iwasaki, Y (2021) | some concerns | some concerns | low | low | low | |
