# Supplementary material for: Is neoadjuvant chemotherapy followed by surgery the appropriate treatment for esophagogastric signet ring cell carcinomas? A systematic review and meta-analysis
Source: Front Surg. 2024 May 6;11:1382039. doi: 10.3389/fsurg.2024.1382039 (PMC11102960; doi:10.3389/fsurg.2024.1382039)
Supplement: Supplementary file 3 [file Datasheet1.docx]

1. **Search strategy for Web of Science and Central:**

**Web of Science:**

TS = (“Signet Ring Cell” OR “Signet Ring Cells” OR “Signet Cell”)

AND TS = (cancer OR carcinoma* OR adenocarcinoma* OR neoplas* OR tumor OR tumors OR tumour* OR malignan*)

AND TS = (neoadjuvant* OR neo-adjuvant OR preoperativ* OR pre-operative OR perioperativ* OR peri-operative OR “followed by” OR following OR induction)

AND TS = (radiochemotherap* OR chemoradiotherap* OR chemoradiation* OR chemotherap* OR therap*)

**Central:**

(“Signet Ring Cell” OR “Signet Ring Cells” OR “Signet Cell”):ti,ab,kw

AND (cancer OR carcinoma* OR adenocarcinoma* OR neoplas* OR tumor OR tumors OR tumour* OR malignan*):ti,ab,kw

OR MeSH descriptor: [Carcinoma, Signet Ring Cell] explode all trees

AND (neoadjuvant* OR neo-adjuvant OR preoperativ* OR pre-operative OR perioperativ* OR peri-operative OR induction OR “followed by” OR following):ti,ab,kw

AND (therap* OR radiochemotherap* OR chemoradiotherap* OR chemoradiation* OR chemotherap*):ti,ab,kw OR MeSH descriptor: [Chemoradiotherapy] explode all trees)

OR MeSH descriptor: [Neoadjuvant Therapy] explode all trees OR MeSH descriptor: [Induction Chemotherapy] explode all trees
