# Supplementary figures and images for: Is neoadjuvant chemotherapy followed by surgery the appropriate treatment for esophagogastric signet ring cell carcinomas? A systematic review and meta-analysis
Source: Front Surg. 2024 May 6;11:1382039. doi: 10.3389/fsurg.2024.1382039 (PMC11102960; doi:10.3389/fsurg.2024.1382039)

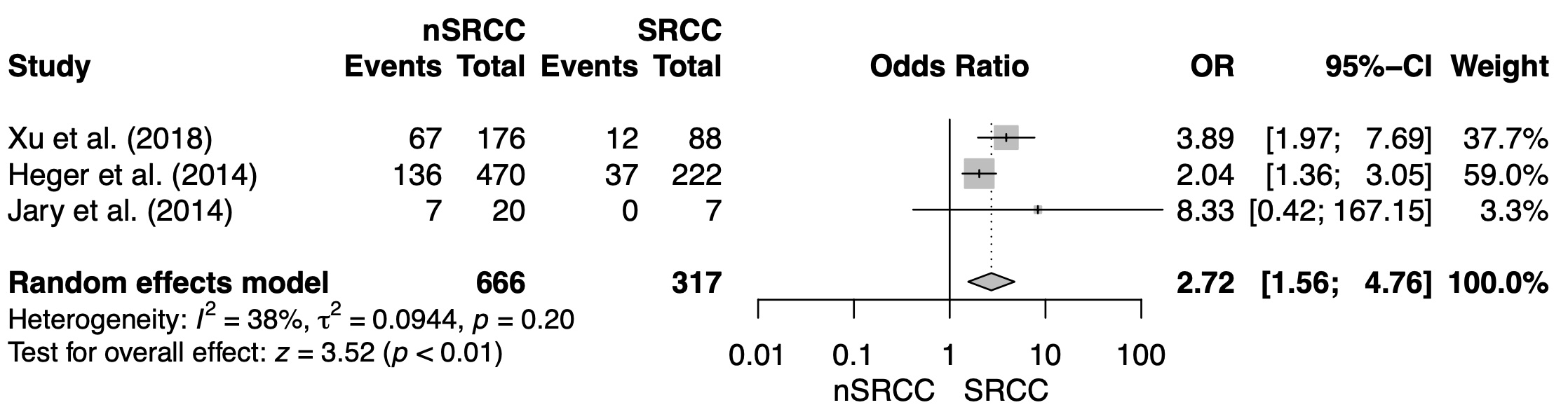

Supplement: Supplementary file 4 [file Image1.jpg]
